# Perceptions and attitudes of Rohingya community stakeholders to pregnancy termination services: a qualitative study in camps of Cox’s Bazar, Bangladesh

**DOI:** 10.1186/s13031-024-00574-9

**Published:** 2024-03-04

**Authors:** Atiya Rahman, Joe Strong, Pragna Paramita Mondal, Audrey Maynard, Tasnima Haque, Ann M. Moore, Kaosar Afsana

**Affiliations:** 1 BRAC James P Grant School of Public Health, BRAC University, Dhaka, Bangladesh; 2 London School of Economics and Political Science, London, UK; 3 Guttmacher Institute, New York, USA

**Keywords:** Rohingya, Pregnancy termination, Abortion care, Menstrual regulation, Bangladesh

## Abstract

**Background:**

Rohingya women suffer from inaccessibility to sexual and reproductive health services in Myanmar. After the forcible displacement of the Rohingya from Myanmar to Bangladesh in 2017, pregnancy termination services have been increasingly important and desired, while knowledge gaps and obstacles to access services still exist. The role of community stakeholders is critical as gatekeepers and decision-makers to improve and strengthen pregnancy termination services for women in camps. However, there is paucity of evidence on their perspectives about pregnancy termination. This qualitative study aims to understand the perception and attitudes of Rohingya community stakeholders to pregnancy termination in the camps of Cox’s Bazar.

**Methods:**

We used purposive sampling to select 48 participants from the community stakeholders, 12 from each group: majhis (Rohingya leaders), imams (religious leaders), school teachers, and married men. We conducted in-depth interviews of all the participants between May-June 2022 and October-November 2022. Data were coded on Atlas.ti and analysed using a thematic content analysis approach.

**Results:**

Multiple socio-cultural and religious factors, gendered norms and stigma associated with pregnancy termination acted as barriers to women seeking services for it. From a religious stance, there was greater acceptance of pregnancy termination in the earlier period than in the later period of pregnancy. We observed that pregnancy termination among community stakeholders in earlier stages of pregnancy than later. However, circumstances, such as a woman’s marital status, whether she sought her husband’s permission or her ability of childcare capacity, were often framed by community stakeholders as ‘acceptable’ for pregnancy termination. Health concerns and social and contextual factors can influence community stakeholders supporting pregnancy termination.

**Conclusions:**

The community stakeholders perspectives on barriers and enablers of pregnancy termination were variable with the context. These perspectives may support or impede women’s ability to choice to seek pregnancy termination services. To improve women’s choice to pregnancy termination, it is critical to consider roles of community stakeholders in creating their supporting attitudes to women’s choice and access, and to designing targeted culturally appropriate interventions with communities support and engagement.

**Supplementary Information:**

The online version contains supplementary material available at 10.1186/s13031-024-00574-9.

## Background

Women and adolescent girls are among the most vulnerable people in precarious situations in humanitarian settings [1, 2]. Sexual and reproductive health (SRH) complications, such as unwanted pregnancy, induced abortion, and obstetric complications, are more common to these women than to women in non-humanitarian populations [3]. Up until the 1990s, reproductive healthcare received a disproportionately low priority from humanitarian organizations. During the International Conference on Population and Development, Cairo, reproductive health was (re)framed as fundamental human right. Despite this, studies report that in humanitarian settings, a significant number of women cannot access safe abortion services [1, 4]. Where these services are not available, women seek less biomedically safe abortion care, which can increase the risks of post-abortion complications [5]. Access to abortion within humanitarian settings is limited by significant structural barriers, including the legality of care and willingness of health organizations to provide services and the costs associated with care in contexts where people have little access to economic resources [6, 7].

Rohingya women live in one of the most densely populated and largest displacement camps in the world in Cox’s Bazar district, Bangladesh. They face significant vulnerabilities including barriers to accessing adequate SRH services [8–10]. The Rohingya are a Muslim ethnic minority group from the Rakhine State of Myanmar and have experienced decades of persecution, deprivation, internal displacement, and statelessness in their own country [11]. For a long time, the Rohingya people have been severely and systemically oppressed, with limited freedom of movement, limited access to food, health care, education and job possibilities [12, 13]. While in Myanmar, they faced extreme inequality and injustice with the highest poverty exacerbated by sexual violence [14]. Nearly a million Rohingya have become forcibly displaced Myanmar nationals (FDMNs) and now reside in camps of Bangladesh, with extremely difficult living conditions [15–17]. Women constitute 52% of this Rohingya population [18]. In 2018, UNFPA’s findings indicated that there were around 325,000 women of reproductive age among the FDMN Rohingya population, accounting for 27% of the overall population at that time [18]. Moreover, there remains a significant need for SRH services among Rohingya women who are of reproductive age in the camps [19].

In camps of Cox’s Bazar, with the coordination of health sectors and SRH working groups^1^, primary and secondary health care services including SRH and family planning (FP) are being provided by key implementing partners [20, 21]. There is a provision for menstrual regulation (MR) and post-abortion care (PAC)^2^ services within the camps of Cox’s Bazar [22], however, MR is distinctly defined to be distinguished from abortion care^3^. In Bangladesh, abortion is illegal except for the purpose of saving a woman’s life following the penal code of 1860 [23]. The penal code uses the term ‘miscarriage’ instead of ‘abortion’ to refer ‘termination of pregnancy’ [24]. Since 1979, the government of Bangladesh allows a procedure known as MR^4^, to regularize menstruation in a state of non-pregnancy [23].However, in the context of Rohingya camp the word pregnancy termination is used by the Rohingya community to make it synonymous with MR [25].

Sexual and reproductive health and rights (SRHR), including access to and information on the laws and regulations and policies, vary widely among countries. In Bangladesh, knowledge of MR varies [26] though more than 70% women heard the term [27]. It is rarely common that the displaced or migrants populations have adequate knowledge of host countrie’s laws, policies, and services regarding abortion care. Since SRH services are available and accessible, structural barriers are less of an issue in Rohingya camps [28]. Among Rohingya communities living as FDMN, factors such as a lack of knowledge about SRH services and their availability, paired with a lack of acceptance of pregnancy termination due to religious beliefs, lack of trust, linguistic barriers and less interest in FP services can act as barriers to access these SRH services [29]. Family planning methods are available with a slow uptake shaped influenced by community based socio-cultural and religious norms and influence of camp activities [30].

An individual’s access to abortion care is nested within the influences of their broader community and structural environments. Women negotiate their SRH through complex social realities including their loss of social networks and unfamiliar cultural, linguistic spaces [31]. However, the community-level norms, social and religious expectations and stigma surrounding pregnancy termination are linked to women’s decisions regarding where to seek SRH services and the level of secrecy they maintain to navigate these norms [32, 33]. Community stakeholders can shape social norms around the acceptability of pregnancy termination and therefore, the potential outcomes [34, 35]. These stakeholders are embedded in patriarchal gendered systems that create conditions for men to dominate reproductive decisions [36, 37]. Among forcibly displaced Rohingya communities, evidence illustrates how men act as community gatekeepers over SRH decisions, early marriage, fertility decisions, and women’s mobility [10, 35, 38]. There is a paucity of evidence on the attitudes of these community stakeholders towards pregnancy termination. Such evidence is necessary to better understand potential barriers that women might face to making decisions and accessing care on pregnancy termination.

Where SRHR access and acceptability are deeply rooted in gendered socio-cultural and religious norms [39–41] understanding key community stakeholders’ attitudes and norms towards pregnancy termination among Rohingya women is essential. Although a growing body of research has explored the complexities of pregnancy termination services in the camps from the point of view of experts and health service providers [26, 39, 42], no qualitative research has empirically investigated it from the community stakeholders perspectives. This paper aims to understand the perceptions and attitudes of Rohingya community stakeholders to pregnancy terminations, including barriers and facilitators that women face to access such services in camps of Cox’s Bazar.

## Methods

### Study design

This qualitative study is part of a larger mixed-method research project that also included community-based survey (CBS) with married women of reproductive age and health facility survey (HFS) with healthcare providers/managers. We collected qualitative data from specific population within the Rohingya community to interrogate the study objectives.

### Study settings

As of February 2023, over 957,971 Rohingya reside in 33 camps scattered across Ukhiya and Teknaf upazilas (functioning as an administrative sub-district/sub-unit of a district) of Cox’s Bazar district of Bangladesh, a coastal region in the Southeastern part of the country [43]. All camps have a high population density, which ranges from 40,000 inhabitants per square km [44]. Among the 33 camps, we purposefully selected four camps (camp 2W, 8W, 14 and 15) from Ukhiya upazila. The selection of these four camps was based on their ratio of facilities that provide SRH services to women of reproductive age group. Camp 2W and 15 have a higher ratio, whereas camp 8 and 14 have a lower ratio. The common scenarios of these study sites are shown in Table 1. Across all camps, overcrowding and poor living conditions have a detrimental impact on the health and wellbeing of the population.

### Study participants and sample

**Table 1:** Household and population wise distribution of study sites

Aspects	Information in numbers			
	Camp 2W	Camp 8W	Camp 15	Camp 14
Total Households	5336	6648	11384	6844
Total women (12 to 59 years old)	7985	10130	17390	10769

The study population for this qualitative study were selected to represent a range of key groups from Rohingyas living in the camps: majhis^5^, imams^6^, teachers, and married men. These research participants were found to play a role in shaping and influencing interpersonal and community-level norms and attitudes toward pregnancy termination (Table 2).

Participants of each community group selected for interviewing were purposively sampled using snowball sampling. This was accomplished by asking people in the community to suggest who would be knowledgeable and interested in discussing about unintended pregnancy and pregnancy termination.

While we conducted a pilot study in November 2021, one of the major learning was that the Rohingya participants did not have a common understanding of the term ‘MR’. The majority of the participants considered MR as pregnancy termination. As a result, we used ‘pregnancy termination’ or ‘ending a pregnancy’ in our interview to make them understand about MR services [25].

Majhis were approached to facilitate the identification of potential married men and imams within each research site. Bangladesh government camp officials employed by the camp authority also assisted in the identification of teachers. In total, 48 participants were interviewed for the study. The sample size was equally distributed in the four camps. Three participants from each category were selected from every camp. The sample size was small with no significant variation in the characteristics among the participants in each camp (Table 1).

### Data collection

Two male anthropologists and one female public health researcher were hired as interviewers. They were supported by two male and one female interpreters recruited from the Cox’s Bazar region and were fluent in the local Chittagonian language. Chittagonian is the indigenous dialect spoken in the regions of Cox’s Bazar where the camps are situated. Similar strategies were followed in other studies to collect data on the Rohingya population [45, 46]. According to a language assessment conducted by the Translators without Borders, the Rohingya languages and Chittagonian dialects share 70–90% similarities [47]. In addition, the interpreters had skills and experiences in conducting qualitative interviews in Rohingya camps, and were very much acquainted with camp scenario. Two researchers from the core research team trained both the interviewers and interpreters for over ten days on interviewing techniques, rapport building and interview guides and facilitated a discussion on the camp health systems. Both interviewer and interpreter were trained together to interview the participants fluently without compromising the meaning of the questions. All the tools used for interviews were developed by the research team and pretested in camp 3, which was excluded from the data of the main study sites. The tools were adjusted based on the feedback from the interviewers (additional file 1 and 2).

**Table 2:** Rational behind choosing the study participants

Participants from Rohingya community	Selection criteria
Married men	Rohingya married men play a significant role in household decision-making.
Teacher	Teachers play a crucial role in instructing and directing Rohingya students and their families, which helps foster social cohesion and community development.
Imam	Imams are religious leaders in the community who serve as both prayer leaders and important sources of SRH information and religious teachings for Rohingyas.
Majhi	Majhis were influential men who were appointed to run a specific geographic block within a camp.

A pair of male interviewer-interpreters conducted interviews with male participants while a pair of female interviewer-interpreters conducted interviews with female participants respectively. With the participants verbal consent prior to data collection, the in-depth interviews (IDIs) took place in their preferred location, maintaining as much privacy as possible. The places participants chose to be interviewed were their own homes, mosques, and campsite management offices (inside camps). Some participants spoke Bengali or Bangla (Bengali and Bangla are two terms of the same language, however here we used the term Bangla to refer to the Bengali population who are living in Bangladesh and West Bengal, India), which interviewers could also speak fluently. All the interviews were recorded on electronic recording devices. Transcriptions into Bangla for interviews in Rohingya/Chittagonian were done the next day, followed by the English translation. Out of 48 interviews, eight were inaudible, so the transcripts were incomplete. Hence, we carried out an additional eight interviews, two from each group, during October to November 2022. The duration of the interviews ranged from one hour to one and a half hours.

### Data analysis

The software Atlas.ti version 23 was used for coding and analysis, through preliminary analysis began during the data collection. All field team members discussed the findings after returning from the field each day. The research team performed an initial review of the transcripts to check the quality of the interviews. In the data cleaning phase, the Bangla transcriptions were further checked against the audio recordings to confirm accuracy. In addition to the research team, quality checking was also conducted by a Rohingya translator for the accuracy of the translation from the audio recording to the English translation.

We used thematic analysis to analyze the data. While reviewing the transcripts, the research team generated the initial code book by using inductive and deductive coding. Five members of the research team coded five transcripts to ascertain intra-coder agreement. Then, the codebook was revised based on this agreement. At the same time, themes were created, reviewed, and named by re-reading the transcripts. Once all the data were coded, the team created metrices. The quotes were used to give voice to the community and reinforce the interpretation or explanation in the narratives.

## Results

### Socio-demographic profile of the study participants

The socio-demographic profile of the participants is described in Table 3. Among the 48 participants, 17 were over 40 years, 14 were less than 30 years and the rest between 31 and 39 years. Majority of them were male (46 out of 48) and two were female. Thirty-nine participants had some formal education under the Myanmar National Curriculum whereas 14 attended primary school (grade 2–5), 24 attended secondary school (grade 6–10) and one was a University graduate. Most of the imams held the same occupation while in Myanmar. However, all majhis and 10 male teachers had diverse occupational backgrounds in the past, engaged in farming and fishing. Currently, some reported receiving a fixed amount of money from different NGOs and local government institutions to execute their responsibilities as community leaders.

**Table 3:** Study Participants characteristics

	Variables	Frequency (*n* = 48)	%
Participant’s Age (mean = 37)	less than 30	14	29
	30 to 39	17	35
	40 and more	17	35
Participant’s Gender	Male	46	96
	Female	2	4
Participant’s Current Occupation	Imam	12	25
	Majhi	12	25
	Teacher	12	25
	Unemployed	10	21
	Others (Fisherman, Day-laborer)	2	4
Participant’s Current Income (BDT*)	Salary 2500 to 10000	33	69
	Humanitarian aid**	14	29
	No fixed income	1	2
Participant’s education	Secondary and more	23	48
	Primary	10	21
	Islamic	12	25
	No education	3	6
Occupation in Myanmar	Business & Farming	25	52
	Imam and *Hujur*	10	21
	Student	6	13
	Teacher	4	8
	Housewife	2	4
	Community Chairman	1	2

*Note: BDT 1 = 0.0076 Pound sterling

** The food, materials and logistics that are received by the Rohingya people from different INGOs like UNHCR refers humanitarian aid. No liquid money is provided as humanitarian aid in the camps

### Community understanding of pregnancy termination

The stakeholders who participated in the study had varied levels of knowledge regarding pregnancy termination and available services. All the participants had a clear understanding of what pregnancy termination meant to them. Many of them were familiar with the words “*fua fori giui*” meaning termination of pregnancy on its own (spontenous abortion) and “*fua felay diui*”meaning to destroy the foetus (induced abortion/pregnancy termination) respectively. Again, pregnancy termination was also viewed as ending a pregnancy, which is the ‘murder of an unborn child’ or ‘intentional killing of a child’. Some heard about pregnancy termination services available in camps from the imams of the mosques.

Most participants were not familiar with the term MR. During our initial visits to the camps, we used the term ‘*mashik jari*’ (English equivalent is period regularization) to make the term, MR more comprehensible. However, the majority of the participants considered MR and pregnancy termination synonymous, describing them as the ending of pregnancy or pregnancy termination. Few participants had a clear understanding of the procedure of MR which loosely referred to as getting the period back by taking medicine and terminating the pregnancy by taking medicine. As one of the religious leaders mentioned,

“Yes, I know that. If this is the case, taking medicine will make you menstruate again. But MR has no Rohingya equivalent term in their dialect. Here most of them buy medicine from the pharmacies and take it to complete their work”[33 years old imam, camp 2W].

### Religious norms and acceptability of pregnancy termination

Participants emphasized how their attitudes towards pregnancy termination were deeply shaped by religious teachings, and indicated the important role that imams could have on disseminating particular views that in turn shaped the acceptability of pregnancy terminations.

Religious norms shape and influence attitudes and practices in relation to pregnancy termination. The significance of religion was emphasized by most of the participants across all groups, particularly how it affected their mindsets and stances towards pregnancy termination. Pregnancy termination was impermissible in Islam, as perceived by the majority of the participants. We found that religion had a negative impact on the acceptability of pregnancy termination. Imams (religious leaders) who were influential in the camps specifically cited that religious doctrine never supported pregnancy termination.

“[Imams] are the people of God. We must follow the Qur’an and Hadith [a group of oral traditions that record the prophet Muhammad’s sayings along with descriptions of his everyday life [the Sunna], which together serve as the main source of Islamic law outside of the Holy Qur’an]. I think it is bad according to the Qur’an and Hadith. (33 years old Imam in camp 2w)

The concept of ‘sin’ and the ‘soul’ was often referenced to relation to pregnancy termination by the participants. Some explained that conception or the birth of the baby was decided by God, not by humans. Thus, taking medications for pregnancy terminations was considered as a sin because it put an end to a life, and humans would remain accountable to God for ending a life.

However, religious beliefs were not singularly against pregnancy termination. Many respondents attitudes towards pregnancy termination shifted depending on how they conceptualized the pregnancy at different stages of gestation. In particular, many respondents discussed the concept of ‘ensoulment’ as a process that occurred around three months after conception. The acceptability of pregnancy termination was witnessed when it occurred relative to this period of ‘ensoulment’. One imam stated:

“Then the life of the fetus starts at the three months of conception. According to Islamic law, it is a great sin to terminate the baby after that. Termination is the same as killing. (45 years old Imam in camp 8w)

Various opposing views prevailed among imams about the interpretation of pregnancy termination in Islam. Religious doctrine influenced the imams negative attitudes to pregnancy termination to a great extent. On occasions, the reality of women’s situation compelled them to support pregnancy termination. However, their positive perspective was equally guided by religious construal.

Thus the views of community men were shaped by the words of the religious leaders, which they also practiced in their daily lives. As mosques are regarded as a place of trust and unity, the community sometimes relied on what religious leaders said after prayer time. Community leaders and men’s attitudes linked to the timing of pregnancy termination was correspondingly developed by religious discourses largely cited by imams from the Qur’an or Hadith. Participants also indicated that religious leaders made it explicit that certain pregnancy termination was permissible at specific times of the pregnancy period. As such, married men used similar language around ensoulment and explicitly illustrated how this shaped their attitudes towards pregnancy termination.

“[MR/pregnancy termination service] is good because this is done before the soul of the child begins. In such a case, by getting the menstrual regulation service, the blood clot is removed from the womb with menstrual blood, and thus the fetus is aborted. *Huzur’s* [religious leaders] said this [MR] is permissible in Shari’ah.” (38 years old married man in camp 8w).

Here the respondent indicated that their attitude was informed by their religious leaders, who made explicit that certain types of pregnancy termination at certain times were permissible. Like this respondent, community stakeholders attitudes towards pregnancy termination were influenced by the religious interpretations of the imams from the Holy books.

### Gendered norms and pregnancy termination

Deeply rooted in cultural and social traditions, gender norms predominantly dictated the expected roles of women, emphasizing their identity as mothers, wives, and daughters. When it came to decisions about terminating pregnancies, women often did not have the final say. Instead, the decisions depended on either their husbands or other family members.

Gendered norms that determine the acceptability of women’s sexuality and behavior had a significant role in influencing attitudes towards pregnancy termination. Participants described the social expectations on Rohingya women to stay inside the home taking care of their families.

This view was most frequently vocalized by imams and majhis. For participants, expectations around women were constructed on their fertility and role as mothers, and pregnancy termination was framed as antithetical to this:

“Married people do not do these things because they want to have children. Everyone wants to have a baby. If she has a baby in her womb, there is no question of termination” (29 years old imam in camp 15).

Due to the social and reproductive expectations on women, pregnancy termination services from health facilities were not deemed by imams and majhis as acceptable.

However, whereas the imam above did not consider married women needing pregnancy termination services, other participants operationalized alternative gendered narratives in their views. Married men emphasized the role of husbands and immediate family members to provide permission to married woman seeking pregnancy termination services.

“If a married woman wants to have a pregnancy termination, first she has to get permission from her husband, then she has to get permission from her parents, father-in-law and mother-in-law” (33 years old married man in camp 14).

This common response reflects the local socio-cultural and gender norms pertaining to how women are expected to behave when it comes to their reproductive and sexual health and decision-making for healthcare. The husband’s involvement was important in the pregnancy termination of his spouse. Social acceptance hinges on marital status. While women were seeking abortion care, community people perceived that the women had no issue in terminating a pregnancy as the husband’s permission and consent had already been obtained.

“[The community] does not say anything to [married women]. They would just say, ‘Why is pregnancy termination necessary? There would be many sins [if you abort]’. They didn’t say anything about them because they have a husband.”(25 years old teacher in camp 14)

Social acceptance of pregnancy termination was instantaneously established for married women with the husband’s consent, even though the issue of committing sin still prevailed. Furthermore, unmarried pregnant women who sought pregnancy termination care were expected to obtain a family’s approval. Seeking healthcare services for pregnancy termination without involving family could lead to significant negative consequences on women, regardless of their marital status. This included conflicts within the family as well as forced disclosure of pregnancy termination to the community. Among the community stakeholders, teachers believed that unmarried woman should consult the family members while seeking pregnancy termination care to obtain their family’s support when needed.

“The family members do not say anything if [an unmarried girl] takes the services after consulting with them. If she does not consult with them then it becomes a huge problem. The situation worsens when the community people get to learn about her situation” (23 years old teacher in camp 8w).

Gendered norms created conditions that framed women for their ability to conceive. Pregnancy termination, therefore, was positioned as antithetical to these expectations. The role of husbands and family members was potentially critical for women navigating pregnancy termination services and helping mitigate some negative social outcomes. This does, however, compound a husband or family member’s power over a woman’s decision-making, embedding women in gendered cultural systems in which they navigate permission and/or secrecy.

### Pregnancy termination and negative attributes ascription to women

The study participants revealed how society judged women for opting for pregnancy termination, taking into account their social status. The community’s perception of pregnancy termination varied, with both negative and, in certain circumstances, positive.

The stakeholders in our study frequently used stigmatizing language and framings of pregnancy termination. Community termed women and girls who terminate their pregnancy as ‘*onasti badi*’ meaning disgusting women; ‘*fowa feloni*’ meaning woman who kills her child or terminates her pregnancy; ‘*fet bajoni*’ meaning woman who carries illegitimate child. Alongside, having already negative attitudes towards pregnancy termination, participants were particularly stigmatizing women who had multiple pregnancy termination:

“Here you are talking about two women. Both had abortions here, so both of them are bad. I don’t think either is good. But, the one who has had a five-time abortion is much worse. Moreover, the ‘*gazab*’ [wrath] of Allah will fall upon her.” (35 years old majhi in camp 8w).

Regardless of the number of times, committing termination of pregnancy itself was regarded as a ‘sin’, explicating that women who did pregnancy termination would not be able to refrain from the wrath of the almighty God.

Unmarried women and girls also faced particular stigma, which intersected with stigmization of non-marital sex. Many religious leaders and community shared their views against sexually active unmarried women and girls who terminated pregnancies and they used negative labels to stigmatize those women and girls. Their negative perception often labelled these girls as “bad and devil women” in society. Premarital sex was not acceptable in the Rohingya community and was considered as a‘sin’ as much as used for pregnancy termination. The imams reacted to premarital sex and pregnancy termination among unmarried women as completely forbidden in the religion. One imam expected the community to ostracize these unmarried girls and make them suffer.

“If any unmarried people need [pregnancy termination] services, then it is sure that they engaged in ‘*haram kaj*’ [English equivalent is forbidden act - specifically indicating premarital sex here]. *Haram kaj* is strictly forbidden in our religion and has severe punishment for it. So, if they have done it, then they are meant to suffer” (33 years old imam in camp 2w).

Several community leaders acknowledged that stigmatization of unmarried women and girls sought pregnancy termination services at facilities further away from their localities to maintain secrecy and confidentiality,

“Unmarried women can get care after going to hospitals. They hardly go because if anyone sees her, she will fall into problems. She will be treated as bad girl and will not even be able to get married. That’s why unmarried women do not go to hospitals but rather visit the medicine shops for care”. (45 years old majhi in camp 14)

Unmarried girls’ sexual relationships remained hidden from their family members due to social and religious restrictions. The community leaders shared that families usually compelled unmarried girls to terminate their pregnancy for the fear of social ostracization and the potential issues that a pregnancy could mean for their daughters’ future marital prospects. Even though women were perceived to have committed a ‘sin’ and wrongdoing by having sex and getting pregnant out of wedlock, the community leaders had a dilemma about women’s rights to access pregnancy termination care as a human right. To navigate this, many of the participants felt that unmarried women should have access to pregnancy termination services to bring their “monthly period back”. These services were found justifiable for unmarried girls for the avoidance of health problems, unwanted pregnancy, social embarrassment, and future prospects due to their religious stigma around premarital sex and parenthood. The community leaders, especially the majhis, showed a supportive outlook towards women to advise them to seek out safe pregnancy termination services.

“For unmarried women/ girls, these services (MR) are very important. those who are not having periods monthly, you must assume that either the women or girls are having some physical problem or may be due to pregnancy. If anybody comes to me to get advice for those services, then I would tell them to go to the hospital and to discuss the matter with the doctors”. (54 years old majhi in camp 15)

Vilifying attitudes towards pregnancy termination among community stakeholders were particularly prevalent in circumstances of where women were unmarried or had terminated pregnancy before. This included the belief that women who had sought pregnancy termination services should face negative repercussions for their actions.

### Conditionalities supporting pregnancy termination

A woman’s social status was a significant factor influencing a positive view of pregnancy termination. Additionally, situations where terminating a pregnancy was seen as necessary to save a woman’s life were also considered as an ‘acceptable’ reason for a pregnancy termination by participants.

Women’s marital status and some conditionalities influence how the community accepts unwanted pregnancies and pregnancy termination. Community stakeholders, including majhis, teachers, and married men, noted some conditions where pregnancy termination was deemed acceptable included when married women had young and too many children, suffered physical ailments and/or was separated or divorced from their partner. In this context, one participant explained why they supported pregnancy termination when having frequent pregnancies adversely affects the health of the mother:

“Because of pregnancies, a lot can happen in the future. A mother especially could face many difficulties in the future. Mothers are getting sick due to having kids continuously one by one, she is more likely to die. That’s why if it is already in the womb, it should be terminated.” (35 years old majhi in camp 15).

Community stakeholders reiterated that pregnancy termination was justified in saving the pregnant woman’s life. Amongst them, teachers shared their views on when women should access pregnancy termination services. Women who had young children could access these services easily to avoid difficulties they might have faced while supporting their family. The participants mentioned that women raising young children or facing higher risk pregnancy could seek pregnancy termination services:

“I think it is better for married people. When a child is small or young, it becomes problematic if another child is born. If someone take these services, they will not face these problems”. (38 years old married man in camp 8w)

The community’s perception of pregnancy termination was often negative. Participants were more accepting of the need for PAC in instances where it might save the woman’s life. Here, one married man commented on PAC services:

“Indeed, she should get post-abortion service. If somebody is sick, she should get services. Illicit relationships are a disgusting crime, yes, but to save her life, she needs treatment." (40 years old married man in camp 8w)

It is quite evident that in the situation that threatens women’s lives, the stance and mindset of men also change and support women’s healthcare seeking, which nuances the less supportive religious and gendered norms towards pregnancy termination.

Rohingya communities faced significant inequalities and injustices while in the camps. The participants spent much of their time worrying about how to make it through the harsh conditions and the future of their children. Referring to these terrible living conditions, the majhis expressed that women were not being criticized by the community as much as before the displacement if they sought services for pregnancy termination now. To meet their own basic needs, people were occupied with varied daily activities. Despite receiving food rations, shelters, health care and some education, lack of money and the future of the next generation were the major concerns expressed by the community stakeholders. Given this context, the majhis considered pregnancy termination as an option from the financial perspective of maintaining a family.

“I think [MR/pregnancy termination] is good because my wife did not suffer at all. If the child had been born, I would not be able to raise him/her well. It would have become difficult for me as I do not have that much financial solvency.” (60 years old majhi in camp 2w).

The community stakeholders reasoning in support of pregnancy termination were aligned with the conditions of women living in the camps that made pregnancy termination services acceptable within their community.

## Discussion

The Rohingya crisis has put women in an environment where they have access to pregnancy termination services as denoted by the community, and continue to face barriers to seeking these services. Understanding the complexity in displacement camps necessitates deep exploration of the socio-cultural context that women navigate. This study is among the first that took an in-depth qualitative approach to explore the Rohingya community stakeholders perceptions of pregnancy termination, social norms, and family involvement, and how they see their roles in women’s access to pregnancy termination services. It illustrated, the community stakeholders perceptions towards pregnancy termination services and the sociocultural factors that legitimized them. Their perceptions of these elements were important in understanding the SRH environment of the camps because of their individual key roles in facilitating access to pregnancy termination services and their own attitudes to pregnancy termination constructed by the collective norms that shaped women’s access.

This study demonstrated the mixed and negative perceptions toward pregnancy termination services among key community members, which were especially informed by their religious beliefs. The connection between attitudes and religion reflected in other studies conducted in humanitarian settings and others where women are stigmatized due to perceived religious laws [48, 49]. Specifically, imams who are highly respected in the Rohingya community considered pregnancy termination as largely unacceptable. Given the critical role of community leaders in shaping the broader normative environment around SRHR in many countries in Sub-Saharan Africa and South Asia including Kyrgyzstan, Ghana, Peru, Nairobi, Kenya [37], the stigmatizing views of imams can have a significant effect on community-level attitudes towards women and girls who seek MR/pregnancy termination services due to the fact that they exert a significant impact on both individual and collective conduct. Though most participants believed pregnancy termination to be forbidden under religious doctrine, some people in other settings often think of MR and pregnancy termination as different depending on the duration of pregnancy [50, 51]. The terminology between MR and pregnancy termination have different acceptance among the people in the Rohingya community. For many participants, perceived religious beliefs were driven by the perception that a fetus gets ‘soul’ or life at the three months of gestation. This critical juncture of religious beliefs of the ‘ensoulment’ of the fetus led to the permissibility of pregnancy termination services prior to three months of gestation despite varied viewpoints among Rohingya community stakeholders. The findings conformed with other studies and academic literature revealed that pregnancy termination performed during the first 12 weeks of gestation was permissible according to religious doctrine [52]. According to most Islamic scholars, an embryo is considered to be without a soul and hence lacks the full rights of a living person. It is only after being granted a soul that it is recognized as an individual with full rights. However, even without a soul, the embryo is still considered to have some rights, albeit they are limited. According to Islam, it is generally believed that the embryo starts its existence after the soul is infused into it, which occurs 120 days after fertilization [52, 53]. Reproductive health counselling should include information on the availability of safe pregnancy related services, especially the different care options available up to three months gestation, to provide information that would be relevant and culturally acceptable.

The study also highlighted the community’s perception of societal norms, in which the acceptability of a woman’s decision to seek pregnancy termination care depends on whether her family has been involved. To mitigate where pregnancy termination was perceived negatively, in the Rohingya community, husband/parental permission was considered important by participants. Rather than pregnancy termination being framed as a woman’s choice, participants routinely made clear that they expected husbands and in-laws particularly to be involved and sanction any pregnancy or pregnancy termination-related decision made. In the South-Asian context, family members and male relatives are expected to regulate women’s health decisions [54, 55]. As a result of the patriarchal standards that prevail in the Rohingya community, Rohingya women faced obstacles in the decision-making process due to their lack of independence to access services within the camps [56].

Our findings showed that perceived acceptable conditions surrounding pregnancy termination were related to the mother’s health risk, having more or very young children, undesirable camp living conditions and an unhealthy relationship with the partner. For married women, there was an expectation that the husband’s permission would be given before accessing services and that this would potentially mitigate some stigmatizing community responses to the service. Change in pregnancy termination related perception and behavior is critical for the Rohingya community when the issue is emotional and sensitive [35, 56]. This indicates that this relationality implicitly and explicitly impacts the ability of women to navigate care on pregnancy termination, particularly their ability to make their own decisions without coercion or outside involvement.

Abortions can be highly stigmatized and fraught with emotion, shame, and fear for women, their families, and their partners [57–59]. The data demonstrated community members’ perceptions that unmarried women would face significant stigma, with participants also expressing stigmatizing views towards those women. The high population density and close proximity between families and neighbors exacerbate the ability of a girl or woman to seek care privately or in secret [60, 61﻿]. This intersects with ongoing complexities around accessing safe abortion care among Rohingya women living in camps [19, 62]. Moreover, unmarried women were perceived to have limited information about the accessibility and availability of MR services. Many participants made clear that unmarried women, in particular, should not be engaging in sex or sexually active and that information on sex, sexuality, and reproduction was inappropriate. This exacerbates potential information gaps for particular groups of women who need pregnancy termination services. This also shows that the participants have limited knowledge and understanding that accompanied their views on pregnancy termination. This is compounded by an already mixed level of SRHR knowledge among Rohingya communities [41, 63]. Thus, unmarried women and adolescent girls should also be given information on safe and accessible MR advice in ways that are attuned to the community-level stigma and sensitivities around this information. Knowledge among respondents was often mixed and intersected with religious beliefs, perceptions of the differences between pregnancy termination and menstrual regulation, and stigma. Positive, evidence-based knowledge dissemination of pregnancy termination care, including deconstructing stigmatizing abortion narratives, is important.

Despite the negative attitude towards unintended pregnancy of unmarried women, the community perceived the necessity of access to care. The SRH working group has taken some strategies to improve knowledge and change the community’s attitude toward reducing unintended pregnancies and adopt recommended practices [64, 65]. In that case, behavior changes communication strategies use religion-based elements such as using verses from the religious doctrine. And religious leaders could potentially work as prominent community influencers to bring change. However, studies clearly stated that when the issue is sensitive, the change could be critical [35, 56]. So, it can be assumed that it will be critical to bring about a change in community integration on a sensitive issue like MR or associated care.

### Study strengths and weaknesses

This is the first study on the perception of pregnancy termination with stakeholders from the Rohingya community. The main strength of this study was the inclusion of a variety of participants to explore their perceptions This study focuses specially on the role of key community stakeholders including men, to understand how these populations understand and perceive pregnancy termination and related services. These groups are not exclusively responsible for creating the conditions under which women navigate their pregnancy termination related healthcare. Beyond the scope of this study is the inclusion of other groups (unmarried men, women) who have important roles in care seeking for pregnancy termination. Due to restrictive socio-religious norms, interviews with the participants on sensitive topics act as main challenges of this study. This challenge was addressed by ensuring appropriate term usage during the interview, and avoiding asking questions about participants personal health until later in the interview to ensure a level of respect and trust was first established. Maintaining privacy was of the utmost importance during interviews, not only because of the risk of outsider influence on participants and to maintain confidentiality, but to create a space where participants could speak freely. Even with these protections in place, it must be acknowledged that participants may have withheld or changed certain answers to navigate their comfort levels and privacy during the interview. In addition, since this study involved multiple languages, the process of data collection through the interpreters can be a limitation. This issue of interpreter and interviewer bias was mitigated by reading and re-reading the transcripts and translated notes and listening to audio-records. Core research team members, interviewers and interpreters all participated in the process of reviewing the notes produced by others. Moreover, a skilled Rohingya translator was hired to check and compare sampled English notes with audio-records to confirm consistency and maintain quality and reliability.

## Conclusion

Community stakeholders within forcibly displaced Rohingya communities in Bangladesh had predominantly negative attitudes towards pregnancy termination. In particular, imams used interpretations of religious doctrine to describe pregnancy termination as a sin, and widespread among all respondents were stigmatizing attitudes towards people who had pregnancy terminations, particularly if they were unmarried or had previously had pregnancy termination. There were, however, circumstances described by respondents in which they felt that an pregnancy termination would either be an acceptable decision or a lesser problematic decision. In particular, despite stigmatizing unmarried women for being pregnant, male community stakeholders perceived the decision to seek pregnancy termination as permissible. Moreover, strong gendered norms around husband’s decision-making meant that pregnancy termination were considered less problematic if a husband had provided permission to his wife.

This study can influence the implementation of future MR/PAC programs by assisting national and international actors in humanitarian settings. According to the findings, there are numerous chances to engage with males to increase women’s access to SRHR. In the Rohingya community, where pregnancy termination is stigmatized, culturally appropriate and religion-sensitive information, education and communication intervention, along with access to self-care interventions, might increase women’s autonomy in seeking MR/pregnancy termination services.

### Electronic supplementary material

Below is the link to the electronic supplementary material.

Supplementary Material 1

Supplementary Material 2

## Data Availability

The data that support the findings of this study are available upon reasonable request from the corresponding author. The data are not publicly available due to research ethics board restrictions.

## Footnotes

1 Sexual Reproductive Health (SRH) working group: Led by UNFPA, the SRH working group comprises over 40 partners (representatives from the government of Bangladesh, NGOs, INGOs, and UN agencies providing oversight, identifying, and addressing challenges related to service quality, accessibility, and coverage in SRH in Rohingya camps.

2 Post-abortion care (PAC) Postabortion care (PAC) consists of emergency treatment for complications related to spontaneous or induced abortions, family planning and birth spacing counseling, and provision of family planning methods for the prevention of further mistimed or unplanned pregnancies that may result in repeat induced abortions.

3 Abortion care encompasses management of various clinical conditions, including spontaneous and induced abortion (of both non-viable and viable pregnancies) and intrauterine fetal demise, and also post-abortion care, including management of incomplete abortion.

4 Menstrual Regulation (MR) is a procedure that uses manual vacuum aspiration or a combination of mifepristone and misoprostol to “regulate the menstrual cycle when menstruation is absent for a short duration up to 12 weeks of gestational age.

5 Majhi is the local community leader of Rohingyas. However, they are not a traditional leader nor necessarily respected members of the community.

6 Imam, in Islam, is one who leads Muslim worshippers in prayers and is treated as the head of the Muslim community. Some imams who teach at the Maktab – usually refer hujurs.

## Abbreviations

FDMN: Forcibly Displaced Myanmar Nationals
CBS: Community based study
CIC: Camp-in-Charge
HFS: Health facility survey
IDI: In-depth Interview
MR: Menstrual regulation
SRH: Sexual & Reproductive Health
SRHR: Sexual and Reproductive Health Rights
PAC: Post-abortion Care
