# Supplementary material for: Perceptions and attitudes of Rohingya community stakeholders to pregnancy termination services: a qualitative study in camps of Cox’s Bazar, Bangladesh
Source: Confl Health. 2024 Mar 4;18:19. doi: 10.1186/s13031-024-00574-9 (PMC10910813; doi:10.1186/s13031-024-00574-9)
Supplement: Supplementary file 1 — Supplementary Material 1 [file 13031_2024_574_MOESM1_ESM.pdf]

In-depth Interview guideline for Majhi, Imam, and Teacher

Ice-breaking:

a) Please tell me about yourself and your family.
b) Please tell me about the role you play in this community.

Now, I'd like to ask your perspective on reproductive health issues and their importance to the well-being of this community.

1. **Knowledge about the availability and accessibility and Perception regarding barriers for the Family Planning (FP) Services.**

Availability and Access

Please answer this question based on the current situation of your community/ camp:

a. What do you know about available Family Planning (FP) methods in the camps? How do you come to know about what methods are available? Is that where you hear most things about contraception / menstrual regulation? Is it common for men to get information this way?
b. Who provides these services? How would describe the common traits of a FP provider in this community? [Probe: gender, age, ethnicity, language, training and any other traits] How do these traits the service delivery?
c. How important is it to the people in your camp/community that these providers are female?

Peoples' attitude toward FP services

a. What do people say about FP methods? Have peoples' attitude towards Family Planning (FP) services changed since they first came in the camps? In what ways?
    i. What do you think is responsible for these changes?
    ii. Are there people whose views have NOT changed in these ways? Why do you think that is?
    iii. What do you personally think of these changes? Tell me more about why you feel that way.

Challenges, Barriers, and helping factors

a. What challenges are there in getting and using family planning here in the camps? What keeps people from getting and using family planning when they want to? [Probe: Distance, Time, Cost, Accompaniment, perception, stigma, or any other barrier]
    i. How are these barriers different for groups of people [unmarried women, younger women, men]? How do they deal with that?
b. Is there any factor that contributed to the increase of access to FP services? Why and how do you think it improved access for people?

Suggestions and Recommendations

a. What roles NGOs/Imams/Majhis/CICs/Rohingya doctors are currently playing in ensuring peoples' access to FP services? How can they contribute more? What else they can do? [Probe: What role they can play to improve FP access for people with different marital status and age?]
    i. Do you see yourself having a role in the improvement of FP access in your block / community? How?

2. Knowledge and Perception regarding Management of unwanted pregnancies

Availability and Access to Menstrual Regulation (MR) and abortion services

a. Have you heard about Menstrual Regulation (MR) services? [Probe: What do you know about it? Who provides these services in the camps? Where did you learn from it? Is that where you hear most things about menstrual regulation? Is it common for men to get information this way?]
b. What are the consequences for women if others come to find out she had an MR? Please explain why are these consequences. If they were in Myanmar, would the consequences be different? If yes, how would the consequences then?
c. Who can get MR services and who cannot? Are there people in this camp/community who need MR but aren't able to get it? What makes you think that? *[Probe separately for unmarried women, separated/divorced women, married women, unmarried adolescents]*
d. What do you personally think about Menstrual Regulation (MR)? [Probe: Do you think it is it religiously acceptable? Is it culturally acceptable? If someone came to you in your capacity as (Imam / Mahji / CIC), what would you tell them you think about Menstrual Regulation?
e. How would describe the common traits of a MR provider in this community? [Probe: gender, age, ethnicity, language, training and any other traits] How these traits affect people who need MR?

Peoples' attitude towards MR

a. What do people say about MR services? How have you come to know these opinions? Have peoples' attitude towards MR services changed since they first came in the camps? In what ways?
    i. What do you think is responsible for these changes?
    ii. Are there people whose views have NOT changed in these ways? Why do you think that is?
    iii. What do you personally think of these changes? Tell me more about why you feel that way.

Challenges, Barriers, and helping factors

a. What challenges are there in getting and using MR here in the camps? What keeps people from getting and using MR when they want to? [Probe: Distance, Time, Cost, Accompaniment, perception, stigma, or any other barrier]
    i. What do you think about the cost associated with getting a MR service? Would you identify it as a barrier? Why?
    ii. What do you think about the distance and terrain one has to cover to get MR service? Would you identify it as a barrier? Why?
    iii. What role do you think the religious and cultural practices and prohibition is playing in accessing MR services? Would you identify it as a barrier to receive these services? Why?
    iv. Can you identify any other reason as barriers to access MR? Why do you think of those as barriers? [Probe on lack of female providers and language as barriers]
b. How are these barriers different for groups of people [unmarried women, younger women, men]? How do they deal with that?
c. Is there any factor that contributed to the increase of access to MR services? Why and how do you think it improved access for people?

Suggestions and Recommendations

a. What roles NGOs/Imams/Majhis/CICs/Rohingya doctors are currently playing in ensuring peoples' access to FP services? How can they contribute more? What else they can do? [Probe: What role they can play to improve FP access for people with different marital status and age?]
    i. Do you see yourself having a role in the improvement of FP access in your block / community? How?

Knowledge about access and availability of traditional ways of managing unwanted pregnancies

a. What do you know about women terminating pregnancies in other ways, for example when the pregnancy is too far along for menstrual regulation or they prefer not to go to a health facility? Where would they go? Who provides these services? How much do these services cost? Are there particular groups of people who you think may need abortion services, but aren't able to get them? [*Probe separately for unmarried women, separated/divorced women, married women, unmarried adolescents*]. Why do you think that is?
b. What do you, personally, think about using these services? Is it religiously acceptable? Is it culturally acceptable? If someone came to you in your capacity

as (Imam / Mahji / CIC), what would you tell them you think about Menstrual Regulation?

c. What are the consequences for women if others come to find out she had an abortion?

d. What do people say about managing unwanted pregnancies? Have peoples' attitude towards managing unwanted pregnancies have changed since they first came in the camps? In what ways?

   i. What do you think is responsible for these changes?

   ii. Are there people whose views have NOT changed in these ways? Why do you think that is?

   iii. What do you personally think of these changes? Tell me more about why you feel that way.

e. How would describe the common traits of a provider that provides these services in the community? [Probe: gender, age, ethnicity, language, training and any other traits] How does traits affect people who need abortion services?

3. Knowledge and Perception regarding Post Abortion Care (PAC) Services.

I am interested to know about your knowledge and perception regarding Post Abortion Care (PAC) services women may need to access when they experience miscarriage or complications of unsafe abortion.

Availability and Access of Post Abortion Care (PAC) services

a. What do you know about PAC services in the camps? How do you come to know about PAC? Is that where you hear most things about contraception / menstrual regulation? Is it common for men to get information this way?

b. Where women seek care when they experience miscarriage or complications of unsafe abortion? How do they go? Who provides these services? How much these services cost? Who is able to access these services? *[Probe on unmarried women, separated/divorced women, married women.]*

c. Are the currently available Post Abortion Care (PAC) services sufficiently serve the need of your people? What make you think so?

d. Which women may have the greatest difficulty of accessing these services? *[Probe on unmarried women, separated/divorced women, married women, unmarried adolescents.]*

e. What do you personally think about PAC services? [Probe: Do you think it is it religiously acceptable? Is it culturally acceptable? If someone came to you in your capacity as (Imam / Mahji / CIC), what would you tell them you think about PAC?

f. How would describe the common traits of a PAC service provider in this community? [Probe: gender, age, ethnicity, language, training and any other traits] How these traits affect people who need PAC?

Peoples' attitude towards PAC

a. What do people say about managing unwanted pregnancies? Have peoples' attitude towards MR services changed since they first came in the camps? In what ways?
    i. What do you think is responsible for these changes?
    ii. Are there people whose views have NOT changed in these ways? Why do you think that is?
    iii. What do you personally think of these changes? Tell me more about why you feel that way.

Challenges and barriers of accessing Post Abortion Care (PAC) services

a. What challenges are there in getting PAC here in the camps? What keeps people from getting and using PAC when they want to? [Probe: Distance, Time, Cost, Accompaniment, perception, stigma, or any other barrier]
    i. What do you think about the cost associated with getting PAC service? Would you identify it as a barrier? Why?
    ii. What do you think about the distance and terrain one has to cover to get PAC service? Would you identify it as a barrier? Why?
    iii. What role do you think the religious and cultural practices and prohibition is playing in accessing PAC services? Would you identify it as a barrier to receive these services? Why?
    iv. Can you identify any other reason as barriers to access PAC? Why do you think of those as barriers? [Probe on lack of female providers and language as barriers]
b. How are these barriers different for groups of people [unmarried women, younger women, men]? How do they deal with that?
c. Is there any factor that contributed to the increase of access to Post Abortion Care (PAC) services? Why and how do you think it improved access for people?

Suggestion and Recommendation

d. What roles NGOs/Imams/Majhis/CICs/Rohingya doctors are currently playing in ensuring peoples' access to PAC services? How can they contribute more? What else they can do? [Probe: What role they can play to improve PAC access for people with different marital status and age?]
    i. Do you see yourself having a role in the improvement of PAC access in your block / community? How?

Closing the interview

4. **Is there anything else you’d like to add before we end the interview?**

Thank you so much for sharing your experiences with us.
