# Supplementary material for: Perceptions and attitudes of Rohingya community stakeholders to pregnancy termination services: a qualitative study in camps of Cox’s Bazar, Bangladesh
Source: Confl Health. 2024 Mar 4;18:19. doi: 10.1186/s13031-024-00574-9 (PMC10910813; doi:10.1186/s13031-024-00574-9)
Supplement: Supplementary file 2 — Supplementary Material 2 [file 13031_2024_574_MOESM2_ESM.pdf]

In-depth Interview guideline for Married Men

1. **Icebreaker/introduction**
   a. Tell me about your family and children [**Prompt:** Who are living in your household? How many children do you have? How old are they?]

Thank you for telling me a little bit about yourself and your family. Now, I would like to ask you questions about child spacing and family planning.

2. **Family Planning**
   a. What do you think of when I say "family planning?" What does that phrase mean? What methods do you think of? Where did you learn about this? [Probe: Is that where you hear most things about contraception? Is it common for men to get information this way? How have the source of receiving such information changed since you came in the camps? Do you have a preference for where you would receive information on contraception? What is that?]
   b. How do other Rohingya men view FP? What do they say about it? [**Prompt:** Would you say they have difference of opinion than you? How you came to know that they have difference in opinion regarding this? How this difference was created according to you? What he did after knowing about this?]
      a. If there is no difference in opinion, then, How he came to know that they have a similar opinion? When and where did the discussion take place? Have their opinions changes since then? If their opinion is changed, how/why their opinion changed?
   c. What methods do most people that you know use? How would someone get family planning methods in the camps? Where should he/she go? How much would it cost? Who can get these methods? And who cannot? Generally, who gets the methods (the males or the females)? Is it common among the Rohingya couples using Family Planning methods after coming to Bangladesh? Did things change in the way that people prevented pregnancy when they came to the camps? How did things change?
   d. Are there differences between ways of preventing pregnancy (FP methods) that men like and that women like? What are the differences? Which Family Planning (FP) method Rohingya men prefer the most? Why is that? What is good about those methods? Is there anything that men you know don't like about other methods? What kinds of things?
   e. What would you say are the challenges and barriers of seeking these methods?
   f. Would you say there is any helping factor that might contributed in increasing the access to FP services? What are the factors? Who do you think contributed to this?

Suggestions and Recommendations

   i. What roles NGOs/Imams/Majhis/CICs/Rohingya doctors are currently playing in ensuring peoples' access to FP services? How can they contribute more? What else

they can do? [Probe: What role they can play to improve FP access for people with different marital status and age?]

a. Do you see yourself having a role in the improvement of FP access in your block / community? How?

3. Knowledge and practice of Abortion among Rohingya men

i. Understanding of Abortion

If the respondent said that he had an experience being involved with an abortion, ask him to relate these questions to his experience

a. What you can tell us about abortion? [**Prompt:** What do you think about abortion? What do you know about immediate and long-term effects of abortion? What are the reasons of abortions for female according to Rohingya men? Are the reasons for induced and spontaneous abortion similar? How are they different? [**Probe** about social, familial, physical, financial, security, demographic or any other reasons]
b. Where do you learn about abortion? [Probe: Is that where you hear most things about abortion? Is it common for men to get information this way? How did you feel about it when you first learned about it? How do you feel about it now?
c. What do most of the Rohingya men think and say about abortion? Would you say your opinion about abortion is similar to most of the Rohingya males? Apart from the dominant thoughts, what other opinions regarding abortion exist among the males? What are these opinions? How do you came to know about there opinions? Where and when do these discussion took place?]
d. Would you say Rohingya males opinions about abortion have changed/modified since they migrated to Bangladesh? If yes, why and how do you think these changed? If no, what aspects have prevented their change of opinions?
e. Who are involved in abortion decision in a household? [Probe: Who do women tell if they want to end a pregnancy? What would they tell? What is the role of the head of the household? What's the role of the husband? What is the role of other female relatives? What is the role of the female who wants to terminate the pregnancy in abortion decision? Are there people that women don't involve in their decision to end a pregnancy who you think should be involved in the decision?]
f. Do married women, who have abortions, always tell their husbands? Under what circumstances might a woman not tell her husband about having an abortion? Can you tell us the story? [**Probe:** How the husband reacted towards his wife after hearing this? How his peers reacted to this instance of him not knowing about the abortion? Do you think most of the Rohingya male would react the same way that he did? If not, how would they react?]
g. If a woman has no husband (i.e., she is not married), how does she decide whether or not to have an abortion? Who does she involve in her decision? What do people say about women who get pregnant when they aren't married? What do they say when

unmarried women have abortions? Do men have different opinions than women do about this? Tell me about those differences.

4. **Stigma**

i. Do couples who have ended pregnancies tell anyone? Do they keep it a secret? Do they keep it a secret only form certain people? Why do you think this is?
ii. How do Rohingya men treat a Rohingya women who attempt or completed an abortion? What kinds of things do men say about these women? [**Probe:** Would they treat similarly if that women were their wife? Would they treat similarly if that women were their daughter? Would they treat similarly if that women were their sisters?]
iii. How do Rohingya men treat men whose wives attempted/completed an abortion? How would Rohingya men behave towards someone whose wife completed/attempted abortion? [**Probe:** Do you think their attitude towards the men whose wife completed/attempted abortion has changed after they took shelter in Bangladesh? What do the Rohingya males talk about it?]
iv. How do Rohingya men treat women who have had a miscarriage? [**Probe:** Would they treat similarly if that women were their wife? Would they treat similarly if that women were their daughter? Would they treat similarly if that women were their sisters?]
v. How Rohingya men view someone who have attempted/completed abortion multiple times? [**Prompt:** Who are those women in Rohingya community who attempt abortion multiple times? How would men's attitude be different towards them than to someone who had attempted abortion once only? And how it will be different toward someone who experienced spontaneous abortion? Why so? How do Rohingya men treat someone whose wife attempted/ended pregnancy multiple times]
vi. Do you know about an incident in which a male supported his wife/daughter/sister for getting an abortion? If yes, please tell us the incident. [**Probe:** How did you came to know about it? Who told you? Why do you think that person decided to tell you? What did you think about it? Under what circumstances is it socially acceptable for men to support their wives in getting an abortion? When is it not acceptable?]
vii. How do Rohingya men view having sex before marriage? Does this happen in the Rohingya community? Under what circumstances? How do you feel about it?
viii. Do Rohingya women feel differently about sex before marriage? Why so? How has migrating to the camps have affected people's feelings about sex before marriage? Do you think that people who have sex before marriage are treated differently here in the camps than they would be in Myanmar? Why is that?
ix. How do Rohingya men treat unmarried pregnant women? Would they treat her differently if she was widowed? Divorced? separated? Do you think their behavior towards unmarried pregnant women is influenced by their migration to the Camps? Are their behavior influenced by the identity of the person in question (sex, social identity, status etc.), or it will remain same?

x. How one should label/name these pregnancy and abortion services to reduce/not invoke stigma from the community? Why it is necessary?

5. **Abortion in Myanmar**

If the respondent said that he had an experience being involved with an abortion, ask him to relate these questions to his experience

a. In Myanmar, what do the pregnant Rohingya women do if they did not want to give birth? Please elaborate [**Probe:** Can people get abortions in Myanmar? If yes, how they would do that? Did you know how to seek abortion services there? How much those services cost? Where one would go? Did women need men or anyone else to accompany them to get the services? Do you think of the abortion services available in Myanmar were safe? Are they safe? How you determine whether it is safe or not?]

b. In Myanmar, did you have direct/indirect exposure to an abortion incident? If yes, please describe in detail.

6. **Availability and Access of Abortion services in the camps**

If the respondent said that he had an experience being involved with an abortion, ask him to relate these questions to his experience

a. What abortion services are available in camps? [**Prompt:** Where in the camps one should go to get these services? How much these services cost? Who are the service providers? Who can get these services? Who cannot get these services?]

b. What do you know about pills one can get from a drug seller to end a pregnancy?

1) Tell me about any experiences you've had ever trying to get such pills, or helping someone else to get the pills? *[Probe for the full story: whether this was in Myanmar or Bangladesh, how the drug seller was identified, how much the pills cost, whether the pills successfully terminated the pregnancy, what the entire experience was like.]*

c. What do you know about women terminating pregnancies in other ways, for example when the pregnancy is too far along for menstrual regulation or they prefer not to go to a health facility? Where would they go? Who provides these services? How much do these services cost? Are there particular groups of people who you think may need abortion services, but aren't able to get them? [*Probe separately for unmarried women, separated/divorced women, married women, unmarried adolescents*]. Why do you think that is?

6.1 Barriers, Challenges and Supporting factors

If the respondent said that he had an experience being involved with an abortion, ask him to relate these questions to his experience

a. How difficult is it to get an abortion of any type in the camps? Is it easier in some camps than in others? What makes it so? Why it is difficult in some camps? Is it more difficult for some people to access abortion services in the camps than others? Which people? Why is it difficult for them?

b. Do you feel like there is secrecy around menstrual regulation /abortion? How does this secrecy make you feel?
c. What do you think about the cost associated with getting an abortion? Would you identify it as a barrier? Why?
d. What do you think about the distance and terrain one has to cover to get abortion service? Would you identify it as a barrier? Why?
e. What role do you think the religious and cultural practices and prohibition is playing in accessing abortion services? Would you identify it as a barrier to receive these services? Why?
f. Can you identify any other reason as barriers to access abortion services? Why do you think of those as barriers? *[Probe on lack of female providers and language as barriers]*
g. Comparing when they/you first arrive in the camps and now, how has the barriers and challenges of accessing these services improved? Why is that? What roles Rohingya men have played to ease these barriers?
h. Can you think of any other issue which might have helped people get abortion services when they need them? What are those issues? Why do you think so?

6.2 Suggestions and Recommendations

If the respondent said that he had an experience being involved with an abortion, ask him to relate these questions to his experience

a. What roles NGOs/Imams/Majhis/CICs/Rohingya doctors are currently playing in ensuring peoples' access to MR services? How can they contribute more? What else they can do? [Probe: What role they can play to improve MR access for people with different marital status and age?]
    i. Do you see yourself having a role in the improvement of MR access in your block / community? How?

7. Care after an abortion/miscarriages

If the respondent said that he had an experience being involved with an abortion, ask him to relate these questions to his experience

a. Do women who have had abortions need any medical care afterwards? What do they need? Do they need different kinds of care if they took pills to cause their abortion versus had it done another way? Do women whose pregnancies ended on their own, who had miscarriages, need medical care? What kind of care? [Prompt: Is there any consequences after an abortion? What are those?]
b. Where and how a woman would seek these kinds of care? How much these post-abortion care services would cost? What medical complications one might face if someone did not get these post-abortion care services?

7.1 Barriers, Challenges and Supporting factors

If the respondent said that he had an experience being involved with an abortion, ask him to relate these questions to his experience

a. How difficult is it to get these post abortion care (PAC) services in the camps? Is it easier in some camps than in others? What makes it so? Why it is difficult in some camps? Is it more difficult for some people to access abortion services in the camps than others? Which people? Why is it difficult for them?
b. How do the doctors/nurses, who provide PAC services, treat the Rohingya people? Tell me based on your experience [If the person do not have any experience regarding this, ask him to tell an experience that he heard from the people he knows]
c. Can you think of any other issues which might keep people for getting abortion services when they need them? What are those issues? Why do you think so?
d. Comparing when they/you first arrive in the camps and now, how has the barriers and challenges of accessing these services improved? Why is that?
e. Can you think of any other issue which might have helped people get abortion services when they need them? What are those issues? Why do you think so?

7.2 Suggestions and Recommendations

If the respondent said that he had an experience being involved with an abortion, ask him to relate these questions to his experience

a. What roles NGOs/Imams/Majhis/CICs/Rohingya doctors are currently playing in ensuring peoples' access to PAC services? How can they contribute more? What else they can do? [Probe: What role they can play to improve PAC access for people with different marital status and age?]
    i. Do you see yourself having a role in the improvement of PAC access in your block / community? How?

8. Sexual Violence

i. What do Rohingya men think about the sexual violence in the camps?
ii. Who are the victims and perpetrators? Who are the most vulnerable people for sexual violence in the camps? When are girls and women sexually abused in the camps?
iii. What do you think can be done to protect people from sexual violence in the camps? What role Rohingya men can play in this?

9. Closing the interview

a. Is there anything else you'd like to add before we end the interview?
